# MRI‐based transfer function determination through the transfer matrix by jointly fitting the incident and scattered $B_{1}^{+}$ field

**DOI:** 10.1002/mrm.27974

**Published:** 2019-10-21

**Authors:** Janot P. Tokaya, Alexander J.E. Raaijmakers, Peter R. Luijten, Alessandro Sbrizzi, Cornelis A.T. van den Berg

**Affiliations:** ^1^ Department of Radiotherapy University Medical Center Utrecht Utrecht The Netherlands; ^2^ Computational Imaging Group for MR Diagnostics & Therapy Center for Image Sciences University Medical Center Utrecht Utrecht The Netherlands; ^3^ Department of Biomedical Engineering Eindhoven University of Technology Eindhoven The Netherlands; ^4^ Department of Radiology University Medical Center Utrecht Utrecht The Netherlands

**Keywords:** active implantable medical device (AIMD), EM simulations, RF heating, safety, transfer function, transfer matrix

## Abstract

**Purpose:**

A purely experimental method for MRI‐based transfer function (TF) determination is presented. A TF characterizes the potential for radiofrequency heating of a linear implant by relating the incident tangential electric field to a scattered electric field at its tip. We utilize the previously introduced transfer matrix (TM) to determine transfer functions solely from the MR measurable quantities, that is, the $\left|B_{1}^{+}\right|$ and transceive phase distributions. This technique can extend the current practice of phantom‐based TF assessment with dedicated experimental setup toward MR‐based methods that have the potential to assess the TF in more realistic situations.

**Theory and Methods:**

An analytical description of the $B_{1}^{+}$ magnitude and transceive phase distribution around a wire‐like implant was derived based on the TM. In this model, the background field is described using a superposition of spherical and cylindrical harmonics while the transfer matrix is parameterized using a previously introduced attenuated wave model. This analytical description can be used to estimate the transfer matrix and transfer function based on the measured $B_{1}^{+}$ distribution.

**Results:**

The TF was successfully determined for 2 mock‐up implants: a 20‐cm bare copper wire and a 20‐cm insulated copper wire with 10 mm of insulation stripped at both endings in respectively 4 and 3 different trajectories. The measured TFs show a strong correlation with a reference determined from simulations and between the separate experiments with correlation coefficients above 0.96 between all TFs. Compared to the simulated TF, the maximum deviation in the estimated tip field is 9.4% and 12.2% for the bare and insulated wire, respectively.

**Conclusions:**

A method has been developed to measure the TF of medical implants using MRI experiments. Jointly fitting the incident and scattered $B_{1}^{+}$ distributions with an analytical description based on the transfer matrix enables accurate determination of the TF of 2 test implants. The presented method no longer needs input from simulated data and can therefore, in principle, be used to measure TF's in test animals or corpses.

## 1 INTRODUCTION

The transfer function (TF) has been introduced to characterize the potential for radiofrequency (RF) heating of elongated linear medical implants like pacemaker and deep brain stimulator leads.^1^ The TF is an implant characteristic that describes the scattered electric field at the tip of an implant, where it is most significantly enhanced, as a function of an incident tangential electric field distribution along the trajectory of the implant. For active implantable medical devices, these are located at the electrode poles which deliver the therapeutic currents to the tissue. The TF simplifies numerical computations to predict heating because it decouples the assessment of the local scattered field (requiring submillimeter detailed modeling of an implant) from the determination of the incident field (generated and scattered by relatively large objects like the transmit coil and human body, respectively).

Experimental validation of numerically determined TFs is performed in phantom experiments. Common measurement methods rely on standardized phantom setups^2^, ^3^ and specialized excitation devices and/or measurement probes. A device that applies a confined and localized electric excitation is stepwise repositioned along the length of the implant while the scattered electric field at the tip is monitored. The ratio between the complex electric field at the tip and the complex field at the excitation device as a function of position along the implant gives the value of the transfer function at the location of the excitation. Alternatively, the excitation device and measurement probe swap position: The induced field along the wire as a function of position is measured while the implant is excited at the tip.^4^ Both methods are equivalent because of the principle of reciprocity. In previous work, this principle of swapping excitation and measurement position was used to develop a fully MR‐based method where the implant is modified into a transmit‐receive antenna that is driven at its tip. For this purpose, an RF cable was soldered to the implant and connected to the scanner by a transmit/receive coil interface. Now the current induced in the implant is proportional to the TF and is calculated from acquired MR images.^5^

**Figure 1:**
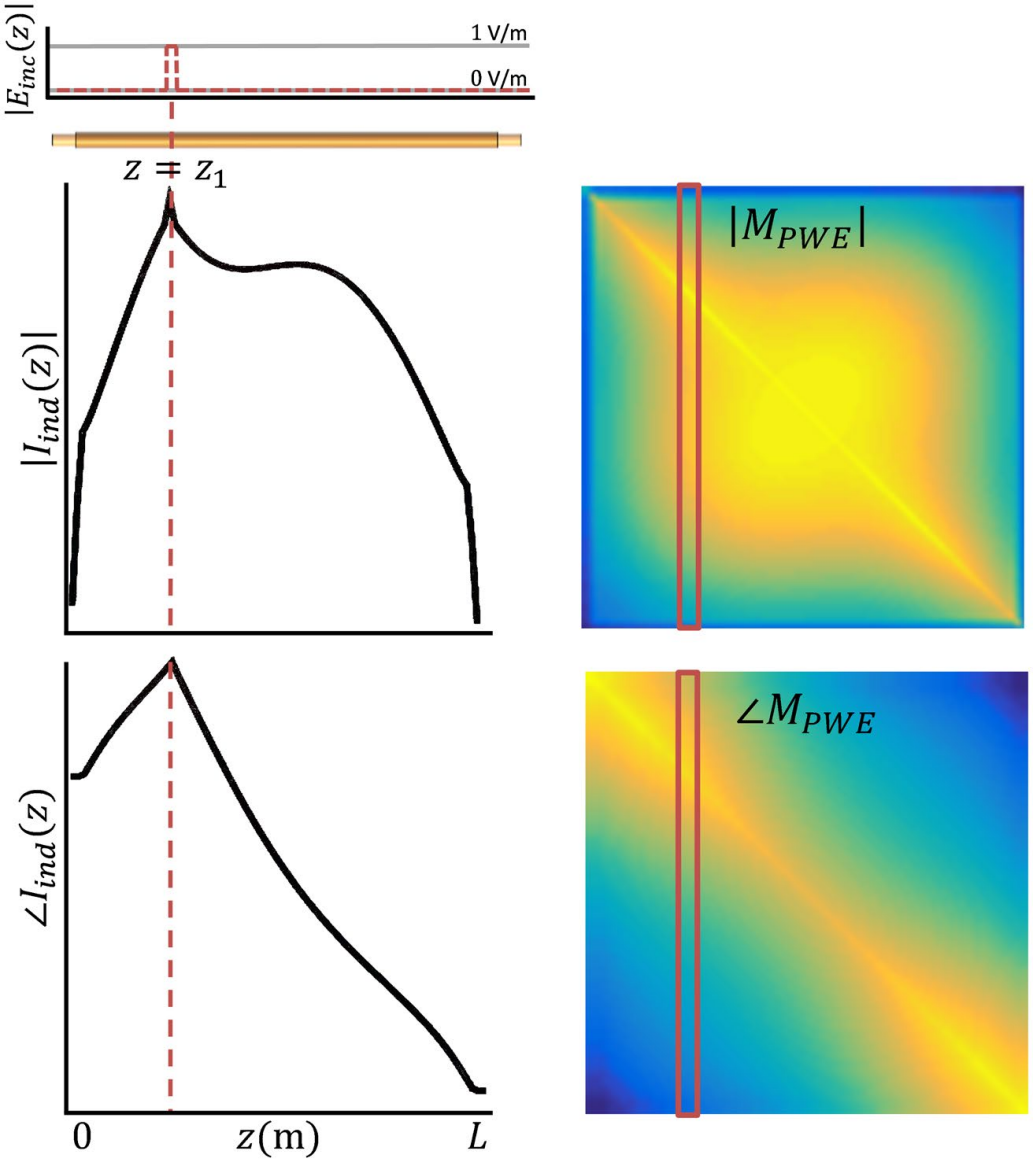
The TM is constructed in simulations by application of a localized incident electric fields of 1 V/m created with 2 plane waves that have constructively interfering electric and destructively interfering magnetic field components. This excitation is repositioned along the entire length of the implant. The resultant current distributions for the various excitations describe the rows of the TM. The width of the excitation determines the resolution to which the TM is resolved. Given an implant with a certain TM and some incident electric field, the induced current in the implant is computed with a matrix multiplication, that is, I = ME

Recently, this method was further improved which enabled the use of standard RF coils and removed the need to make modifications to the implant.^6^ For this purpose, the transfer matrix (TM) was introduced. The TM relates an incident tangential electric field distribution along an implant to an induced current distribution along the entire length of implant. This gives a more complete description of the interaction of an implant with an RF electric field than the TF, which focuses on the description of a single point of worst case heating. In Figure 1 an example of a TM is shown, together with a schematic depiction of the way it is determined in simulations as described previously.^6^ The method for TM determination relies on knowledge of the induced current, that can be measured with MRI,^7^, ^8^, ^9^, ^10^ and the incident electric field, that is determined by simulations.

Even though this works for standardized phantom setups with known or measurable geometrical and electromagnetic properties, it rules out the application on less well‐determined environments where this knowledge is missing as for example in test animals or human corpses, which makes accurate simulations practically infeasible. In principle, if also the incident electric field distribution can be obtained from MRI measurements, in‐vivo transfer function assessment could become possible.

Here we present a method that is able to determine the TF of an implant exclusively from MRI acquisitions in a single phantom experiment. Contrarily to previously presented work the incident electric field is determined from MRI measurements instead of simulations. Under certain, yet realistic, conditions for linear implant safety testing, the z‐component of the electric field distribution is dominant and can be determined from the $B_{1}^{+}$ distribution.^11^ In this work, we present a model describing the RF magnetic field distribution around an elongated implant following an arbitrary trajectory. This model is used to jointly estimate the transfer matrix and the incident background field based on their different spatial dependencies. Firstly, the validity of this model will be tested on simulated $B_{1}^{+}$ distributions for a bare and insulated wire. Secondly, the TM and TF are determined experimentally for the same two wires. Measurements are performed for various trajectories which will result in different incident electric field exposures and induced currents but should yield the same TM and TF. The presented method allows simultaneous TM and TF determination from a $B_{1}^{+}$ magnitude and transceive phase distribution that are assessable with MRI.

## 2 THEORY

Without loss of generality, the $B_{1,tot}^{+}$ distribution in a subject or a phantom with an implant can be written as,

(1) $$B_{1,tot}^{+}=B_{1,bg}^{+}+B_{1,sc}^{+}.$$

In this equation, $B_{1,bg}^{+}$ is the circularly polarized incident background RF transmit magnetic field created in the subject or phantom by the transmit coil that is used for the MR exam. $B_{1,sc}^{+}$ is the circularly polarized component of the scattered RF transmit magnetic field created by the currents that are induced in the implant. The relatively smoothly varying background $B_{1}^{+}$ magnetic field can be accurately decomposed in SPherical And CYlindrical (SPACY) harmonics^12^ that are solutions to the source‐free Helmholtz equation for a homogeneous medium in a spherical and cylindrical coordinate system respectively, that is,

(2) $$B_{1,bg}^{+}\approx \sum_{n=0}^{N_{1}}\sum_{m=-n}^{n}a_{\mathrm{mn}}f_{n}^{m}+\sum_{n=0}^{N_{2}}\sum_{m=0}^{n}b_{\mathrm{mn}}g_{n}^{m},$$

where $f_{n}^{m}=j_{m}\left(\zeta r\right)Y_{n}^{m}\left(\theta ,\;\phi \right)$ and $g_{n}^{m}=J_{m}\left(\zeta \rho \right)Y_{n}^{m}\left(\vartheta \right)$. Here, *j_m_* is the spherical Bessel function of the first kind of order *m*, *J_m_* is the ordinary Bessel function of the first kind of order *m*, and $Y_{n}^{m}$ is the spherical harmonic of order *n* and degree *m*. The tuples (r,θ,ϕ) and (ρ,ϑ,z) describe the spherical and cylindrical (with *z* being the main magnetic field direction) coordinate system respectively with the isocenter of the MR scanner as the origin. The parameter ζ is the complex wavenumber of the electromagnetic waves given by $\zeta =\pm \sqrt{\mu_{0}\varepsilon_{r}\varepsilon_{o}\omega^{2}+i\sigma \omega }$. The Neumann functions are excluded from the solution set of the Helmholtz equation, because they have a pole at the origin and hence are unable to describe the incident fields. The expansion coefficients *a_mn_* and *b_mn_* can be determined by fitting Equation 2 to a measured $B_{1,tot}^{+}$ field distribution with omission of the limited region where the scattered field caused by the implant has a significant contribution. The phase of the complex $B_{1,bg}^{+}$ field is considered to be half the transceive phase.^13^ Note that this transceive phase assumption will not be accurate for $B_{1,tot}^{+}$ because the presence of the implant presents an asymmetric load to both ports of the birdcage. However, our method only requires the transceive phase approximation to be valid for the background field. Given the (near) left‐right symmetry of the setup^14^ and a circularly polarized $B_{1,bg}^{+}$, this assumption will be sufficiently accurate for the experiment presented here because the experiments are performed at 1.5T.^15^

After the $B_{1,bg}^{+}$ is approximated by the SPACY decomposition, the incident electric field can be calculated^11^ because the electric and magnetic fields are intrinsically coupled as described by Maxwell's equations. If one assumes that the longitudinal component of the *B*_1,_ *_bg_* field created by the RF coil is negligible, the z‐component of the electric field is calculated by:

(3) $$E_{z,bg}\left(r;\;a_{\mathrm{mn}}\right)=\frac{1}{\mu_{0}\sigma +i\omega \mu_{0}\epsilon }\left(-2i\frac{\partial }{\partial x}-2\frac{\partial }{\partial y}\right)B_{1,bg}^{+}\left(r;\;a_{\mathrm{mn}}\right).$$

It should be noted that this assumption is valid in the central region and particularly in the midplane of the birdcage coil that are customarily used for 1.5T clinical examinations.

The implant with a random trajectory C, which can be straight or bent and make arbitrary angles with the main magnetic field direction, creates a scattered field. The trajectory C is parameterized by ***r*′**. Therefore, the implant is exposed to a tangential incident electric field, *E_inc_* (***r*′**), that can be computed from this *E_z,bg_* (***r*′**) by multiplication with the cosine of the angle, $\theta^{\prime }$, the wire makes with the z‐axis at ***r*′**, that is,

(4) $$E_{\mathrm{inc}}\left(r^{\prime };\;a_{\mathrm{mn}}\right)=E_{z,bg}\left(r^{\prime };\;a_{\mathrm{mn}}\right)\cos\left(\theta^{\prime }\right).$$

Equation 3 assumes that the x and y component of the electric field are negligible compared to the z‐component of the electric field. The current induced in the implant attributed to this *E_inc_* is calculated through a multiplication with the TM. The scattered magnetic field caused by the implant is subsequently calculated from the induced current using the time‐dependent generalization of the Biot–Savart law known as the Jefimenko equations.^16^, ^17^ These equations describe the electromagnetic field attributed to arbitrary time‐dependent current and charge distributions while taking retardation into account. The magnetic field is given by,

(5) $$B_{1,\mathrm{sc}}\left(r,\;t;\;a_{\mathrm{mn}},\;c_{k}\right)=\frac{\mu_{0}}{4\pi }\int_{C}\left[\frac{I\left(r^{\prime },\;t_{r};\;a_{\mathrm{mn}},\;c_{k}\right){\mathrm{dr}}^{\prime }\times \left(r^{\prime }-r\right)}{{\left|r^{\prime }-r\right|}^{3}}+\frac{\frac{\mathrm{dI}}{\mathrm{dt}}\left(r^{\prime },\;t_{r};\;a_{\mathrm{mn}},\;c_{k}\right){\mathrm{dr}}^{\prime }\times \left(r^{\prime }-r\right)}{c{\left|r^{\prime }-r\right|}^{2}}\right]$$

The scattered field is time and space dependent and can be described by the sets of parameters describing the background field, *a_mn_* and *b_mn_*, and the parameters describing the transfer matrix of the implant, *c_k_*, this equation describes the magnetic field caused by a general time‐varying current *I* running in the implant. It will take a finite amount of time for the field created by the current at ***r*′** to reach a certain position ***r*** which is incorporated in the model through the retardation time $t_{r}=t-\frac{\left|r^{\prime }-r\right|}{c_{m}}$, where *c_m_* is the speed of light in the medium through which the field propagates. Equation 5 is valid whenever the macroscopic Maxwell equations inside a‐ homogeneous dielectric are valid. Because we will assume harmonic time dependency ($B\left(r,\;t\right)=B\left(r\right)e^{i\omega t}$), we can write Equation 5 in phasor notation as,

(6) $$B_{1,\mathrm{sc}}\left(r;\;a_{\mathrm{mn}},\;c_{k}\right)=\frac{\mu_{0}}{4\pi }\int_{C}I\left(r^{\prime };\;a_{\mathrm{mn}},\;c_{k}\right){\mathrm{dr}}^{\prime }\times \left(r^{\prime }-r\right)e^{i\frac{\left|r^{\prime }-r\right|\omega }{c_{m}}}\left[\frac{1}{{\left|r^{\prime }-r\right|}^{3}}+\frac{i\omega }{c{\left|r^{\prime }-r\right|}^{2}}\right]$$

This describes the scattered field attributed to an arbitrarily shaped wire. In the model used here, the wire will be discretized into a number of line segments that correspond to the size of the transfer matrix (e.g., into 40 line segments of 5 mm for the 200‐mm wires considered here). The incident electric field is discretized to the same resolution. In the discretized form after introduction of the TM, Equation 6 can be approximated by a Riemann sum, that is,

(7) $$\begin{array}{cc} B_{1,\mathrm{sc}}\left(r;\;a_{\mathrm{mn}},\;c_{k}\right) & =\frac{\mu_{0}}{4\pi }\sum_{i=1}^{\frac{L}{\left|dr_{i}\right|}}M\left(c_{k}\right)E\left(r_{i}^{\prime };a_{\mathrm{mn}}\right)dr_{i}\times \left(r_{i}-r\right)e^{i\frac{\left|r_{i}-r\right|\omega }{c_{m}}}\left[\frac{1}{{\left|r_{i}-r\right|}^{3}}+\frac{i\omega }{c{\left|r_{i}-r\right|}^{2}}\right]\\ & =\frac{\mu_{0}}{4\pi }\sum_{i=1}^{\frac{L}{\left|dr_{i}\right|}}M\left(c_{k}\right)\frac{-\cos\left(\theta_{i}^{\prime }\right)}{\sigma +i\omega \in }\left(2i\partial_{x}+2\partial_{y}\right)B_{1,bg}^{+}\left(r_{i};a_{\mathrm{mn}}\right)dr_{i}\\ & \times \left(r_{i}-r\right)e^{i\frac{\left|r_{i}-r\right|\omega }{c_{m}}}\left[\frac{1}{{\left|r_{i}-r\right|}^{3}}+\frac{i\omega }{c{\left|r_{i}-r\right|}^{2}}\right], \end{array}$$

which is linearly dependent on (the coefficients describing) the background field. In this equation, *θ_i_* is the angle the *i* ^th^ line segment makes with the z‐axis. The superposition of the circularly polarized RF transmit magnetic incident and scattered field, that is, the total $B_{1}^{+}$ field is hence given by,

(8) $$B_{1,tot}^{+}\left(r;\;a_{\mathrm{mn}},\;c_{k}\right)=B_{1,bg}^{+}\left(r;\;a_{\mathrm{mn}}\right)+\frac{1}{2}\left(B_{1,sc}^{x}\left(r;\;a_{\mathrm{mn}},\;c_{k}\right)+iB_{1,sc}^{y}\left(r;\;a_{\mathrm{mn}},\;c_{k}\right)\right),$$

which is again linearly dependent on the coefficients in the SPACY decomposition. This model can be used to fit a measured or simulated $B_{1}^{+}$ distribution.

Note that Equation 8 describes a complex $B_{1}^{+}$ distribution of which the phase is not experimentally attainable. What can be measured is the so‐called transceive phase, which is the sum of the phase of the $B_{1}^{+}$ and $B_{1}^{-}$ field. For birdcage coils, the phase of the background receive field is (nearly) equal to the phase of the background transmit field.^13^ This is known as the transceive phase approximation stating that the transmit phase will be half the transceive phase. Using this approximation, we can describe the receive background phase with the same SPACY decomposition. A description similar to Equation 8 can be derived for the phase of $B_{1}^{-}$ as a function of the same unknowns. The scattered field contribution to the receive fields is in the direction opposite to the nuclear precession leading to a complex conjugation of the scattered field. Hence, we can write down an expression for the $B_{1,tot}^{-}$ phase, that is,

(9) $$∠B_{1,tot}^{-}\left(r;\;a_{\mathrm{mn}},\;c_{k}\right)=∠\left(B_{1,bg}^{+}\left(r;\;a_{\mathrm{mn}}\right)+\frac{1}{2}{\left(B_{1,sc}^{x}\left(r;\;a_{\mathrm{mn}},\;c_{k}\right)+iB_{1,sc}^{y}\left(r;\;a_{\mathrm{mn}},\;c_{k}\right)\right)}^{*}\right).$$

The transceive phase is the sum the phase given by this equation and the phase of Equation 8. It needs to be stressed that Equation 9 describes the $B_{1}^{-}$ phase in the assumption that the background transmit and receive phase are nearly equal. For our purpose, the decomposition of the magnitude of the receive field is not relevant given that we will only use the MRI‐measurable distributions ($\left|B_{1}^{+}\right|$ and transceive phase).

## 3 METHODS

Equation 8 provides an expression for the $B_{1}^{+}$ field surrounding an elongated implant as a function of the background field parameters *a_mn_* and *b_mn_* and the transfer matrix parameters *c_k_*. If a $B_{1}^{+}$ map is acquired together with a transceive phase distribution, *ϕ_tr_*, with the implant to be tested in a phantom, the transfer matrix can be determined by fitting this expression to the measured (or simulated) field distribution. The corresponding minimization is,

(10) $$\underset{a_{\mathrm{mn}}\in C,c_{k}\in R}{\text{arg min}}\left|\right|\left|B_{1\;meas}^{+}\right|\exp\left(i\phi_{tr,meas}\right)-\left|B_{1\;tot}^{+}\left(a_{\mathrm{mn}},\;c_{k}\right)\right|\exp\left(i\text{arg}\left(B_{1\;tot}^{+}\left(a_{\mathrm{mn}},\;c_{k}\right)*B_{1\;tot}^{-}\left(a_{\mathrm{mn}},\;c_{k}\right)\right)\right)\left|\right|.$$

**Figure 2:**
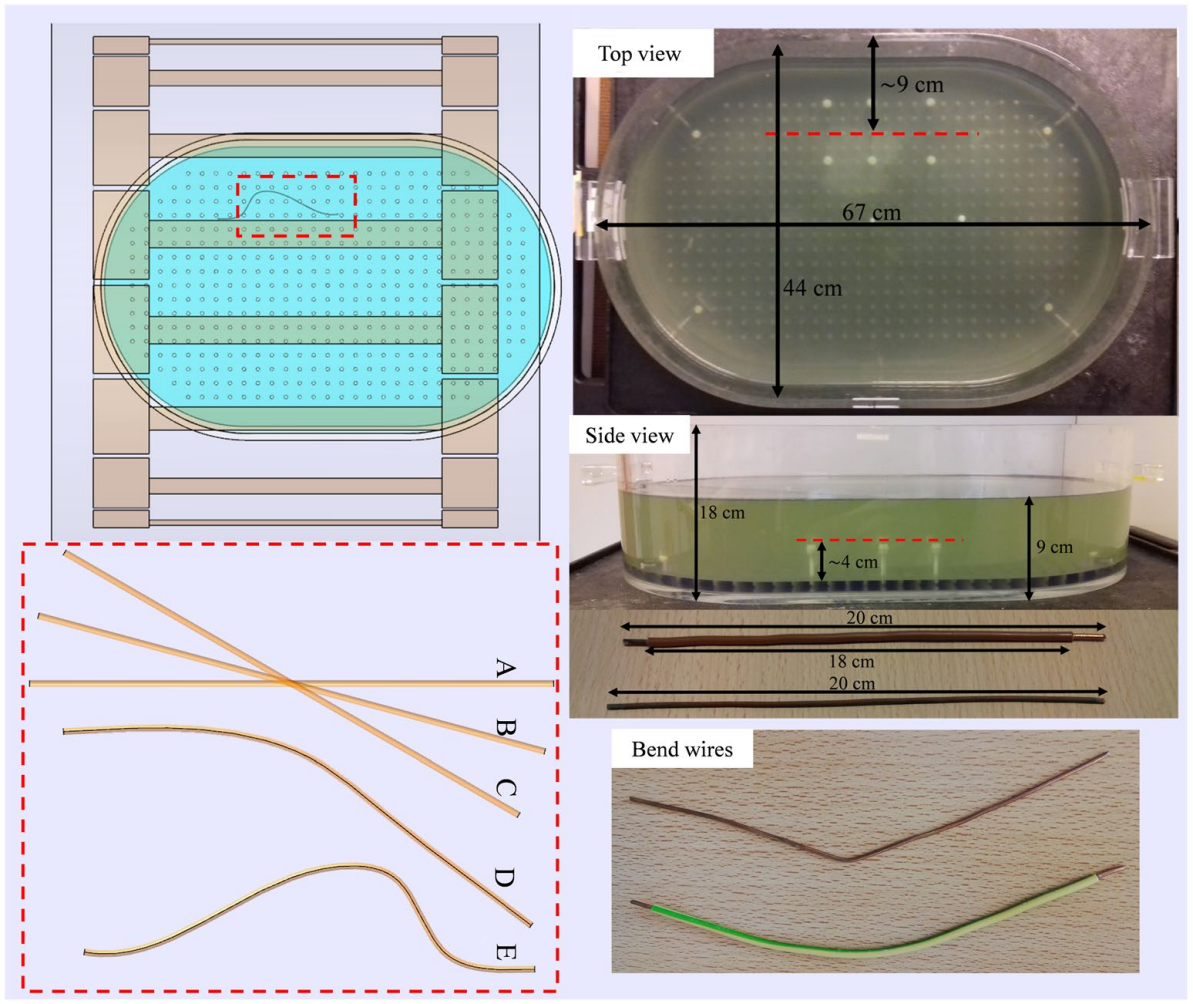
The elliptical ASTM phantom in which the dummy implants are placed is shown on the right. The wires are located on a thin sewing thread stung between 4.5‐cm‐long plastic screws. The wires are positioned approximately 13 cm away from the center of the phantom and submerged under 5 cm of phantom liquid. The setup of the phantom in the birdcage body coil is shown on the left together with the 5 bare wires that are used in simulations

This procedure was tested for 2 implant‐like structures: a 20‐cm bare wire and a 20‐cm insulated wire where the insulation is stripped from 10 mm at both endings as shown in the bottom right corner of Figure 2. For both structures, the MRI measurements are performed with the structures positioned in an elliptical ASTM (American Society for Testing and Materials) phantom also shown in Figure 2. The elliptical phantom has 11‐mm‐thick walls of polymethylmethacrylaat and is filled with hydroxyethyl cellulose gel with relative permittivity of 77 and a conductivity of 0.47 S/m.

The *a_mn_*’s and *b_mn_*'s that describe the background field and determine the incident electric field are found by a least‐squares SPACY decomposition of the complex $B_{1}^{+}$ field disregarding $B_{1}^{+}$ data within a circular region (radius 5 cm) around from the wire. The phase of complex background $B_{1}^{+}$ field is simply considered to be half the transceive phase.^13^ An initial guess for the transfer matrix parameters *c_k_* is obtained by assuming full out‐of‐phase reflection at both endings and a propagation constant based on the permittivity and conductivity of the phantom material (Γ_0_ ≈ −1, Γ*_L_* ≈ −1, *t* ≈ 0.01, $c\approx \sqrt{\mu_{0}\bar{\epsilon_{\mathrm{body}}}\omega^{2}-i\bar{\sigma_{\mathrm{body}}}\mu_{0}\omega }$,). With these initial values, the minimization in Equation 10 is performed. The Nelder–Mead simplex algorithm^18^ implemented in a bound version of MATLAB's fminsearch function (The MathWorks, Inc., Natick, MA) is used to perform the minimization. The *c_k_* that follow from this minimization define the TM and TF of the implant. First, the method is tested in silica, that is, using simulated $B_{1}^{+}$ field distributions. Subsequently, the method is applied for experimental TM and TF determination from measured $\left|B_{1}^{+}\right|$ and transceive phase distributions.

### 3.1 Simulations

The presented procedure and the validity of the field description given by Equations 8 and 9 are tested in silica by numerical FDTD (finite‐difference time‐domain) simulations (Sim4Life; ZMT, Zurich, Switzerland). A harmonic simulation is performed for 50 periods with a –50dB auto termination condition of a model of the phantom shown in Figure 2 in the isocenter of a 1.5T birdcage body coil. A 16 rungs high pass birdcage coil tuned to 64 MHz with 35.2‐cm coil radius and 42‐cm rung length is driven in quadrature mode with 2 voltage sources (IQ‐feed). The RF shield with a radius of 37.2 cm and length of 70 cm is composed of perfect electric conductor. The wire positions and trajectories are also shown in Figure 2. Simulations are performed with a straight wire trajectory aligned with the z‐axis, called A. This straight wire is also placed under an angle of 15° and 30° with respect to the main magnetic field, denoted as B and C respectively. Furthermore, 2 arbitrarily bent wire trajectories D and E are also simulated. For the insulated wire, simulations were performed with implant following trajectories A, B, C, and D, which are labeled Ai, Bi, Ci, and Di respectively.

The resulting $B_{1}^{+}$ magnitude and transceive phase distribution from these simulations are fit with Equations 8 and 9 while optimizing for the TM parameters and keeping the coefficients in the SPACY decomposition of the background field fixed. Note that these distributions ($B_{1}^{+}$ magnitude and transceive phase) are measurable with MRI.

### 3.2 Measurements

The actual MRI measurements were performed in a 1.5T (Philips Ingenia, Best, The Netherlands) MR scanner. $\left|B_{1}^{+}\right|$ distributions are determined experimentally with the variable flip angle (VFA) method.^19^, ^20^ The elliptical phantom is filled with copper sulfate doped hydroxyl ethyl cellulose (9‐cm filling height), with relative permittivity of 77 and a conductivity of 0.47 S/m. The wires are placed approximately 10 cm off‐center in the x‐direction submerged under 5‐cm liquid inside the phantom in the center of the birdcage coil. A straight wire was placed at a 0°, 15°, and 30° angle relative to the z‐axis. Subsequently, the bare wire was bent and an additional acquisition was performed.

The VFA method is used instead of more conventional $\left|B_{1}^{+}\right|$ mapping techniques^21^, ^22^ given that it is able to capture the large dynamic range of actual $\left|B_{1}^{+}\right|$ values in the vicinity of the wire at the expense of increased acquisition time. For this purpose, a collection of 3D spoiled gradient echo images with various nominal flip angles^8^ were acquired. The spoiled gradient echo images had a field of view (FOV; anterior/posterior [AP] × right/left [RL] × feet/head [FH]) of 111 × 430 × 250 mm and a voxel size of 1 × 1 × 5 mm^3^. The relatively high resolution in the AP and RL direction are necessary to capture the rapid decay of the $\left|B_{1}^{+}\right|$ enhancement around the wire. The nominal flip angles were dynamically varied to be 0.25, 0.5,1, 2, 3, 5, 7.5, 10, 12.5, 15, 17.5, 20, 22.5, 25, 27.5, 30, 35, 40, 60, 80, and 100°. The acquisitions had a repetition time of 40 ms, leading to a scan duration of 3 minutes 13 seconds per flip angle. From these acquisitions, the $\left|B_{1}^{+}\right|$ was determined by fitting the signal from the spoiled gradient echo acquisitions on a voxel‐by‐voxel basis as function of flip angle using its well‐known signal equation.^23^ The relaxation times of the phantom fluid were measured^24^ and used for the fit with the signal equation. The resulting fit parameters provide the magnitude of the transmit field distribution $\left|B_{1\;meas}^{+}\right|$ and also the receive sensitivity distribution.

The transceive phase distribution is acquired with two 3D multiecho spoiled gradient‐recalled echo acquisitions with opposite gradient polarities to correct for eddy current contributions^25^ and potential timing inaccuracies.^26^ These scans have the same resolution and FOV as the VFA acquisitions. Four echoes are acquired to correct for static B_0_ phase contributions,^13^ which are dominated by the jump in susceptibility at the interface separating the air and the phantom A simple linear regression on the unwrapped phase data as a function of time is used to correct for B_0_ contributions. The 3D phase is unwrapped using an energy minimization framework based on graph cuts.^27^, ^28^ The B_0_ and eddy current corrected phase only contains the transmit and receive RF phase contributions. This transceive phase and the $B_{1}^{+}$ magnitude distribution are simultaneously fitted with Equation 8 (and Equation 9) to find the parameters {σ_eff_, Γ_0_, Γ*_L_*, *c*_1_, *c*_2_, …, Γ_12_, Γ_23_, …} that describe the TM.

The conductivity of the liquid in the phantom is determined using the SPACY decomposition. The conductivity and permittivity of the phantom medium determine the wave vector of waves that generate the background field. A parameter sweep through realistic conductivity (0:1 S/m) and permittivity (1 *ϵ*_0_:150 *ϵ*_0_) values was performed with steps of 0.01 S/m and 1, respectively, to select the values that give lowest residual in the decomposition of the background field. Subsequently, the SPACY decomposition is performed with these optimal dielectric phantom properties to determine the *a_mn_*’s.

## 4 RESULTS

### 4.1 Simulation

**Figure 3:**
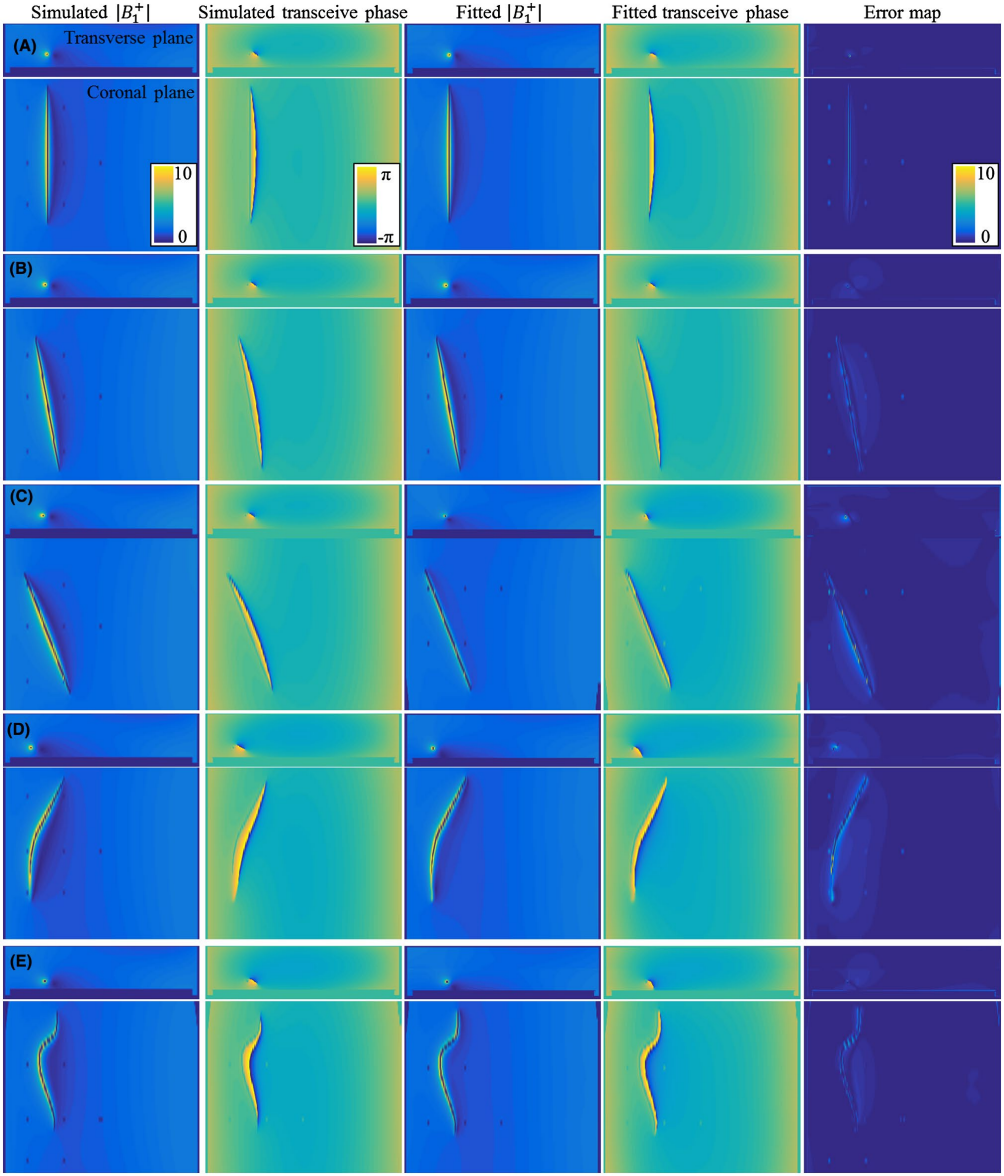
The simulated $B_{1}^{+}$ magnitude and transceive phase distribution around the bare 20‐cm wire in orientations (A), (B), (C), (D), and (E) are shown on the left. These fields are fitted with Equations 8 and 9 using the minimization given in Equation 10. The results from these fits are shown in the third and fourth columns. The absolute error shown on the right is small compared to the $B_{1}^{+}$ magnitude

**Figure 4:**
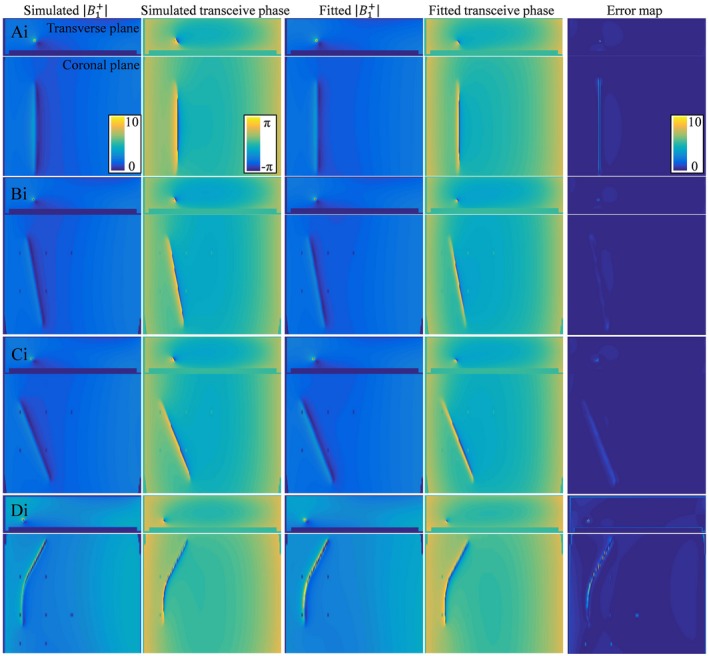
The simulated $B_{1}^{+}$ magnitude and transceive phase distribution around the insulated 20‐cm wire in orientations (Ai), (Bi), (Ci), and (Di) are shown on the left. These fields are fitted with Equations 9 and 10 using the minimalization given in Equation 11. The results from these fits are shown in the third and fourth columns. The absolute error shown on the right is small compared to the $B_{1}^{+}$ magnitude. The screws that keep the implant afloat are not captured by the SPACY harmonics and show up as bright spots in the absolute error map

**Figure 5:**
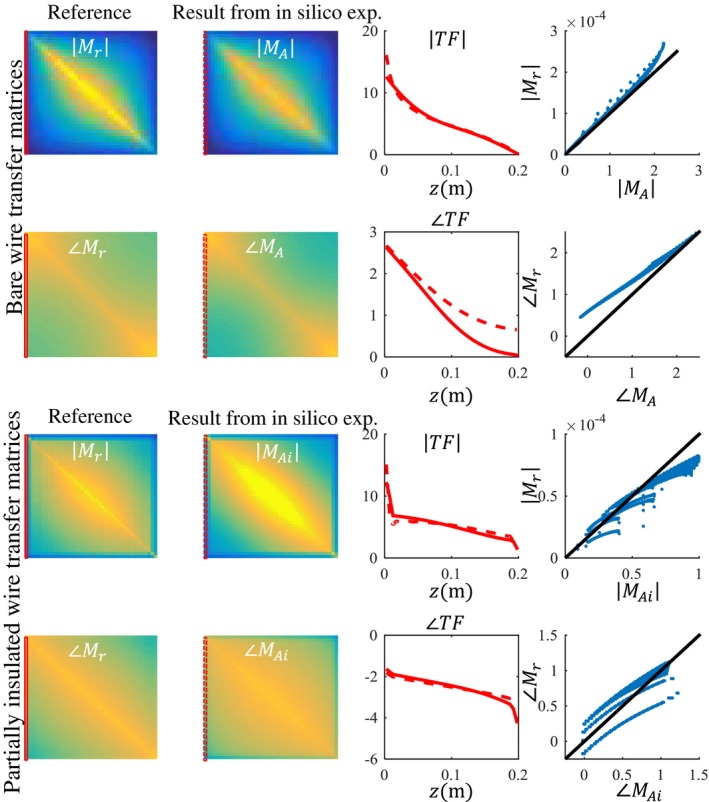
Two examples of the TF and TM that follow from the fit of the fields shown in Figures 3 and 4. The results for the straight wire aligned with the z‐axis are displayed here, that is, from distribution (a) and (ai) in Figures 3 and 4, respectively. Ideally, the TMs that follow from the full field fit should be identical to the reference TMs and lie the line x = y in the outmost right figures. This is not exactly the case, but Pearson correlations are high, with R = 0.983 and R = 0.962 for the bare and insulated wire, respectively

First, the applicability of Equations 8 and 9 to describe the scattered $B_{1}^{+}$ field and transceive phase distribution was tested in silica. Simulated $\left|B_{1}^{+}\right|$ and transceive phase distributions were fitted with Equations 8 and 9 using the minimization given in Equation 10. The results of these fits for a phantom with a 20‐cm bare and insulated wire together with the actual simulated distributions are shown in Figures 3 and 4, respectively. An example of the resulting TM is given in Figure 5 for the straight wire trajectories. The TMs that follow from the other simulated distributions can be found in the supplementary material as Supporting Information Figures S1 to S7. The background parameters are fixed at the values that results from the SPACY decomposition of the total field distributions with data within a 5‐cm distance from the implant removed.

When simulations are available, it is possible to make a comparison between the current that is running in the implant according to the fit and the current that is actually running in the implant from the simulation results. Likewise, the background field that follows from the fit can be compared to the actual background field, which is computed in a separate simulation without a wire in the phantom. The maximal absolute relative error in the current distribution on the wire is 3.8%, and the mean relative error is 1.2% for the straight wire aligned with the z‐axis. The Pearson correlation between the current from simulation and from the fit of the field distributions is 0.9977. The maximal error in the background field estimate was a lot higher with 26.4%, which occurs near the edges of the phantom. Nevertheless, the overall shape of the background field is accurately described with the SPACY decomposition, as indicated by the average relative error of 3.9%. Also, the more distant deviations will not influence the TM and TF assessment. The TF and TM for the bent wire are compared to simulations in Figure 4. The normalized TF that follows from the fit of the $\left|B_{1}^{+}\right|$ and transceive phase distribution closely follows the reference TF. The same holds for the wires in the other trajectories, as is displayed in Figure 5 and in the supplementary material as Supporting Information Figures S1 to S7. These results show that the model accurately decomposes the total field into its background component and the scattered component and hence gives a correct estimate for the induced current and the TF.

For the partially insulated wire, the TM in Equation 8 depends on 10 parameters, which makes the minimization given by Equation 10 somewhat more challenging. The resulting field distribution of the fits for a phantom with a partially insulated 20‐cm wire together with the actual simulated distributions are shown in Figure 4. In the case of the insulated wire, the Pearson correlation between the current from simulation and from the fit of the field distributions is 0.9752 with a mean relative error over the distribution of 2.36% (which is an underestimation of the deviation in the fitted current that is maximal at the transition between the insulated and bare regions). Still, the TFs that follow from the fit are in good agreement with the reference TF for all the trajectories.

**Figure 6:**
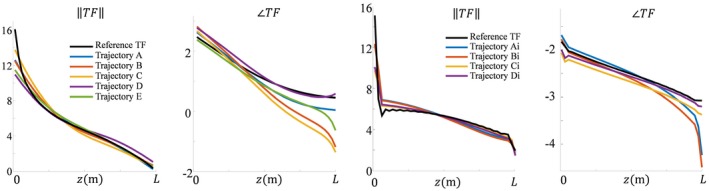
The normalized TFs that follow from the fits of the simulated fields displayed for the bare wires and insulated wires in Figures 3 and 4, respectively. The TFs found from fitting the fields for the different wire trajectories resemble the gold‐standard TF

The results shown in Figures 3 and 4, and the supplementary figures demonstrate that the model given by Equations 8 and 9 describes the fields adequately. The *c_k_* parameters that result from fitting the simulated distributions are the parameters describing the TMs, and thereby the TFs for the bare and partially insulated wire. These TMs are compared to the TMs determined with simulations of subsequently repositioned incident electric fields generated with thin plane wave boxes. The TMs that result from these simulations are considered the ground‐truth reference. For the wires aligned with the z‐axis, the TMs that result from the fit of the field are shown in Figure 5 together with the ground‐truth TM. The first row of the TM is the TF and is shown in the third column of Figure 5. This TF and the TFs that result from the other in silica experiments together with the directly simulated reference are shown in Figure 6.

### 4.2 Experiments

**Figure 7:**
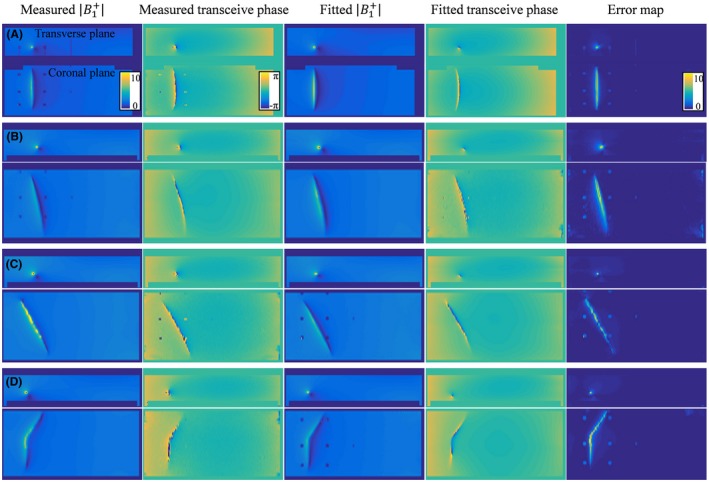
The measured $B_{1}^{+}$ magnitude and transceive phase distribution around the bare 20‐cm wire in orientations (A), (B), (C), and (D) are shown on the left. These fields are fitted with Equations 8 and 9 using the minimization given in Equation 10. The results from these fits are shown in the third and fourth columns. The absolute error is shown on the right. Despite the evident similarity between the distributions, some discrepancies, especially around the wire, remain present

**Figure 8:**
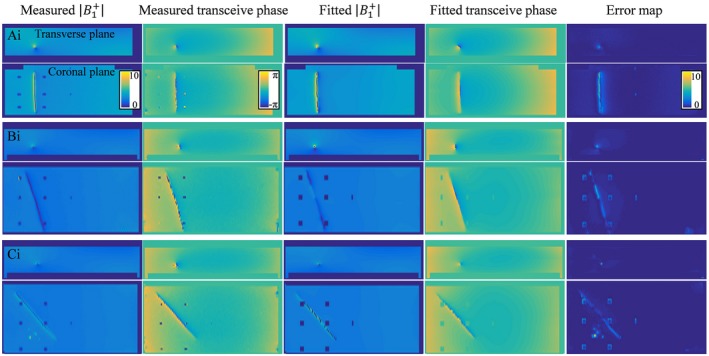
The measured $B_{1}^{+}$ magnitude and transceive phase distribution around the insulated 20‐cm wire in orientations (Ai), (Bi), and (Ci) are shown on the left. These fields are fitted with Equations 8 and 9 using the minimization given in Equation 10. The results from these fits are shown in the third and fourth columns. The absolute error is shown on the right. Again, some discrepancies, especially around the wire, remain present

**Figure 9:**
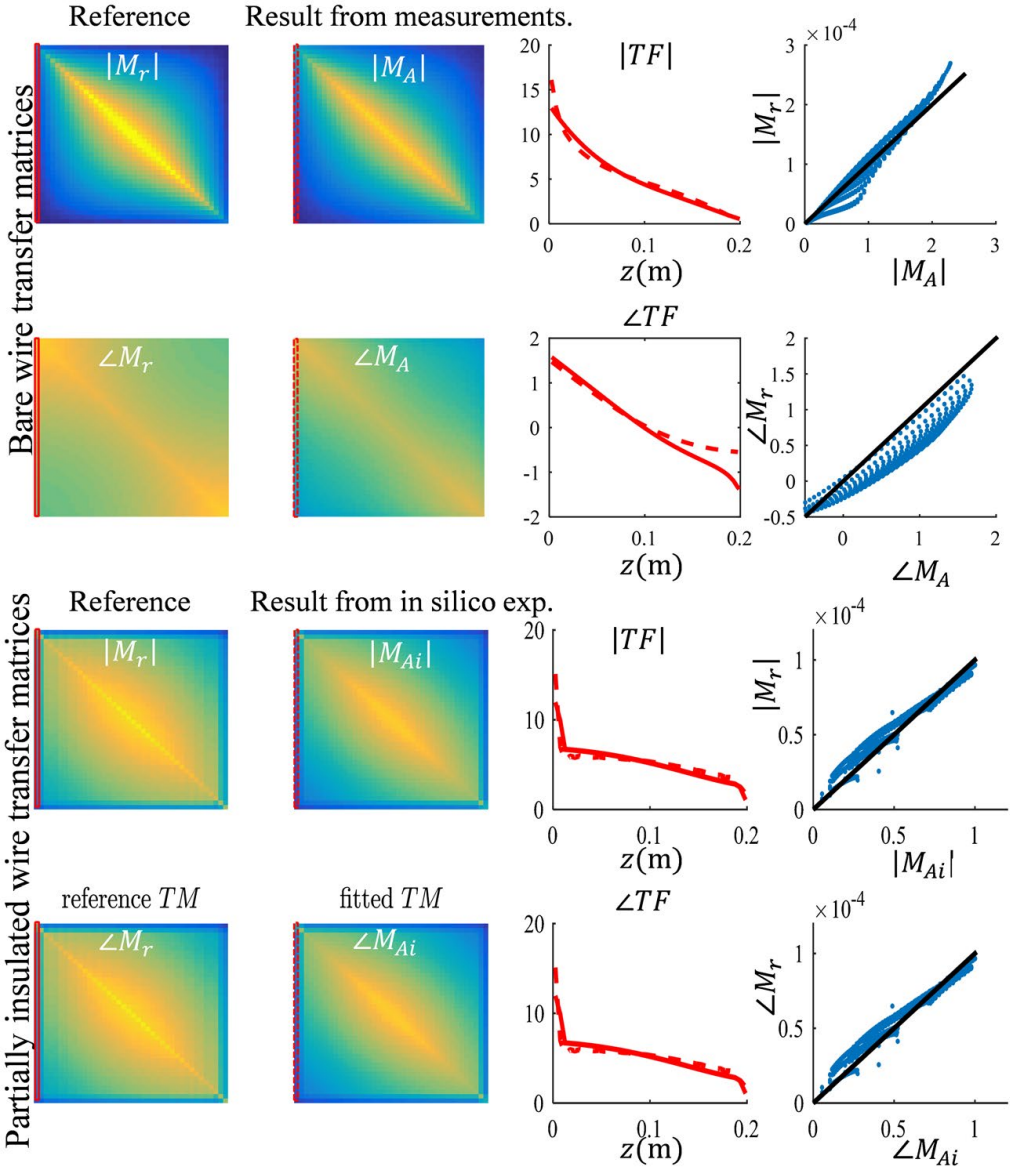
Two examples of the TF and TM that follow from the fit of the fields shown in Figures 3 and 4. The results for the straight wire aligned with the z‐axis are displayed here, that is, from distribution (A) and (Ai) in Figures 3 and 4, respectively. Ideally, the TMs that follow from the full field fit should be identical to the reference TMs and lie the line x = y in the outmost right figures. This is not exactly the case, but Pearson correlations are high, with R = 0.983 and R = 0.962 for the bare and insulated wire, respectively

**Figure 10:**
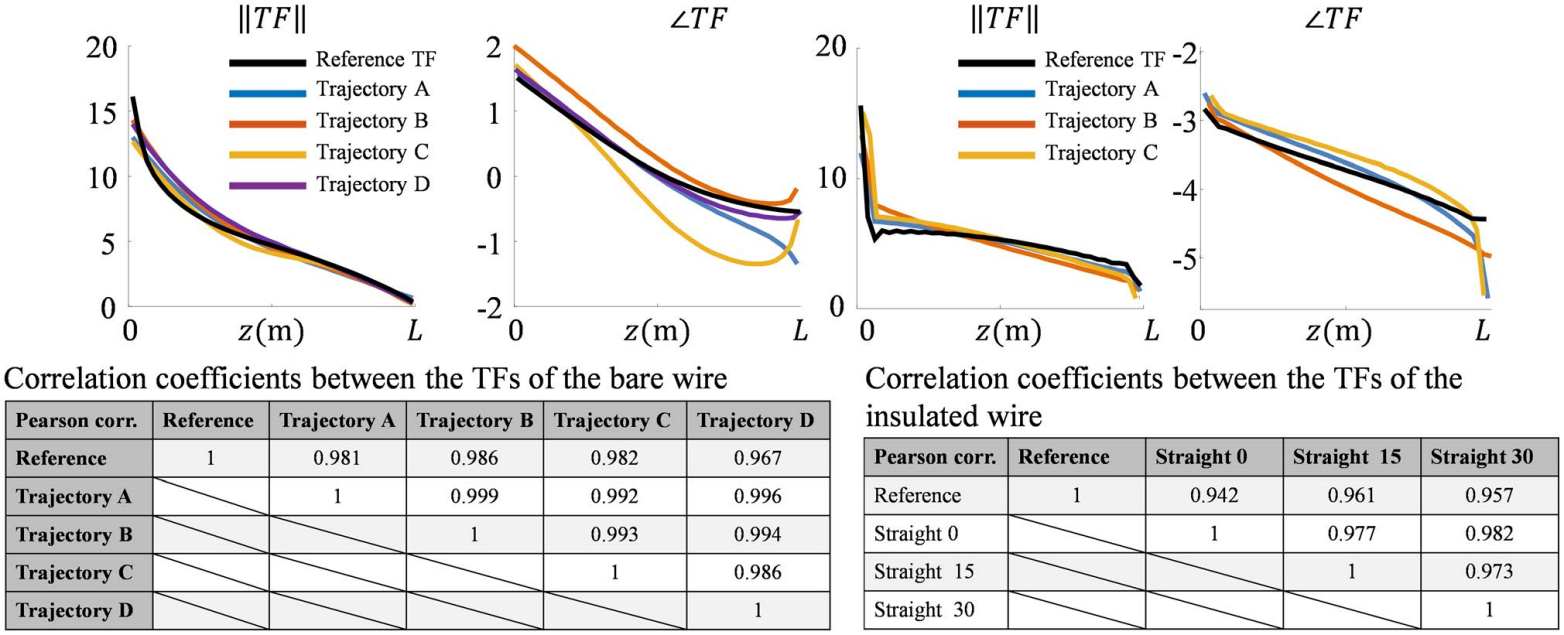
The normalized TFs that follow from the fits of the measured fields displayed for the bare wires and insulated wires in Figures 8 and 9, respectively. The TFs found from fitting the fields for the different wire trajectories resemble the gold‐standard TF and each other. The correlation coefficients are above 0.98 for the bare and above 0.94 for the insulated wires. The agreement found between the TFs can be viewed as a validation of the measurements

The same method is tested on experimentally determined $\left|B_{1}^{+}\right|$ and transceive phase distributions. The measurement results and their fits are shown in Figures 7 and 8, respectively. In the measured data, the plastic screws that are used for the positioning of the wire are visible as signal voids in the $\left|B_{1}^{+}\right|$ distribution. Also, the measured distributions and the relative errors show more grainy distributions attributable to the noise in the MR image. The long wavelength at 1.5T causes the harmonics in the SPACY description of the background field to vary smoothly, which makes the decomposition relatively insensitive to noise, small signal voids, and other abrupt field variations. Therefore, the overall distributions seem to be captured accurately with the fit using Equations 8 and 9 despite the noise. This fit once again provides us with an estimate of the TM and TF. The TMs for the straight wires aligned with the main magnetic field direction are shown in Figure 9. The top subfigures show the TM and its first row (i.e., the TF) for the bare wire and the bottom subfigures show the results for the insulated wire. The TMs for the other orientations can be found as supplementary figures. All the normalized TFs as determined in the various orientations are shown in Figure 10.

## 5 DISCUSSION

A new method has been developed that is able to measure the TF of a medical implant using only MRI experiments. The method is based on the previously introduced TM. The first row of the TM is the TF. Both the TM and the $B_{1}^{+}$ background field are parameterized by a small set of unknowns (~10 and ~40, respectively). This enables the measurement of the TF by acquiring only two MRI data sets that provide the $B_{1}^{+}$ magnitude and transceive phase distributions. The experimentally determined TFs are in good agreement with the reference TMs with Pearson correlation coefficients of R = 0.971 and R = 0.952 for the bare and insulated test implant, respectively. The presented method enables assessment of the TF using only MRI experiments without the need for dedicated hardware, modifications to the implant, or simulations of the setup.

Measurement of the TF by MRI as presented here is only feasible for elongated “piecewise constant” implants, that is, elongated implants that consist of a small number of segments where each segment has constant effective wave propagation properties. The different segments can have different wave propagation constants, similar to the 3‐segment insulated wire parameterization that was used as a test implant throughout the work presented here. Only then is the previously introduced attenuated wave model for the TM applicable.^6^ For more complex implants, the parameterization might become more complex, making the optimization in Equation 10 numerically challenging.

Both results presented in Figure 6 and Figure 10 reveal minor deviations between the normalized TF as determined by the presented method and the reference. Particularly for the in silica experiment (Figures 3, 4, and 5), these deviations might seem remarkable given that the input fields are simulated and do not have any imperfections that are typical for measured field distributions. The deviations likely originate from simplifications that were made for the presented method that are approximately, but not entirely, valid. The most important assumption is the transceive phase approximation required for the background field determination. The phase of the $B_{1}^{+}$ field is not exactly equal to half the transceive phase with deviations ranging from –0.29 to +0.30 radians in simulations. The maximal relative overestimation in the $B_{1}^{+}$ phase distribution compared to half the transceive phase is 16.7%, and the maximal relative underestimation is 12.3%. Nevertheless, the overall distributions are similar, with an average relative error of 3.6% and a Pearson correlation of 0.924. The largest errors occur at the edges of the phantom. Overall, this minor violation of the transceive phase approximation partly explains the modest deviations in Figures 3, 4, 5 given that it will result in inaccuracies in the electric field determined from the derivative of the complex $B_{1,bg}^{+}$.

However, for setups where the transceive phase approximation breaks down, the method in its current form will show larger deviations. The deviation will lead to inaccuracies in the incident electric field determined from the complex $B_{1,bg}^{+}$ using Equation 3. Hence, caution has to be taken when imaging is performed with another transmit coil or when the implant is positioned away from the center of the birdcage coil. The RF interactions of an implant can, however, be assessed in a different imaging location and with a different coil than the actual examination. This freedom should be used to make sure the assumption underlying Equation 3 is accurate.

Second, the assumption that the z‐component of the RF magnetic field is negligible, that is, the electric field is fully oriented in the z‐direction, might not always hold. In the phantom experiment presented here, on average 80.3% of the magnitude of incident electric is contained in its z‐component. At the location of the implant, this percentage is 97.8%. So, the incident electric field can be considered to be z‐directed. This is not generally true and especially starts to fail around material interfaces in the xy‐plane. Both the x‐ and y‐component of the electric field as well as the error in the transceive phase approximation are considered to be responsible for the discrepancy between the reference TFs and the ones determined with in silica experiments are shown in Figure 5.

Obviously, even more discrepancies are visible between the experimentally determined TM or TF and the reference. One reason can be minor differences between the experimental and the simulation setup. For example a potential cause of deviation is the difference in material properties of the insulation layer around the insulated wire, which is considered to be polyvinyl chloride and can have quite a significant effect.^29^

The choice for the multi–flip angle $\left|B_{1}^{+}\right|$ mapping technique is based on previous work.^8^ This is a time‐consuming method (3 minutes 13 seconds per flip angle for 20 flip angles, leading to a total acquisition time of 1 hour 4 minutes 20 seconds) and requires acquisitions with a high nominal flip angle (100°). Other $B_{1}^{+}$ mapping techniques^21^, ^22^, ^30^ will be faster, but may not have the required dynamic range. Optimization of the $B_{1}^{+}$ mapping sequence is not yet performed and may speed up the presented method. Furthermore, the implants used throughout this work do not generate a susceptibility artifact. Some implants induce B_0_ distortions, especially when they are not aligned with B_0_, which result in distinctive signal voids and blooming artifacts and also require adaptations to make customary B_1_ mapping techniques work.^31^ Especially more bulky implants will require additional effort to make the presented method applicable. For the proof of principle presented here, the variable flip angle (VFA) has shown to be the most suitable $B_{1}^{+}$ mapping technique, but for different experiments, other choices might prove more fitting.

To validate the presented method, an independent second electric field exposure can be used to perform separate measurements and verify whether the same TF is found. Such a different incident electric field distribution is obtained by repositioning the implant in the phantom. The implants in the various trajectories will be exposed to different incident electric fields, and hence various currents will be induced. Nevertheless, the TMs and TFs distilled from fitting the $B_{1}^{+}$ magnitude and transceive phase distributions should be, in principle, the same given that it is generally assumed that different loading conditions have negligible impact on the TM. All measured TFs turn out to correlate strongly.

Conventionally, transfer function is determined in homogeneous, liquid phantoms. Only then is the measurement probe able to scan along the full length of the implant. The method described here is based on MR images, and hence the measurement phantom does not need to be fluid or homogeneous. Therefore, this method bears potential for measuring the transfer function in heterogeneous media, such as inhomogeneous phantoms or even corpses. For this purpose, some difficulties still need to be addressed. One of these is that the attenuated wave model will presumably require a location‐dependent wave propagation constant to describe the attenuation and wavelength of the currents in the implant. Likewise, the SPACY decomposition of the background field might need to be extended with solutions to the Helmholtz equation for inhomogeneous media. However, a recent study shows that even for inhomogeneous situations, the SPACY decomposition seems to work adequately.^12^

In principle, the presented method could even be considered as a stepping stone for the measurement of the TF in vivo. However, for this purpose, even more challenges need to be addressed. First of all, a significant speedup of the measurement is necessary. This would require the adaptation of the method such that it can use a local receive array, rather than the birdcage body coil, for receive as presented here. Furthermore, a fast, low‐power B ‐mapping technique with large dynamic range would be needed. If in vivo TF determination becomes possible, certain worst‐case assumptions could be relaxed, possibly enabling scanning of patients with implants of which the RF heating potential is unknown or has resulted in a contraindication for MR exams. MR‐based TF determination could furthermore lead to faster scanning or improved image quality in the case of subjects with MR conditional implants.

## 6 CONCLUSION

A new method has been developed that is able to measure the TF of a medical implant using only MRI experiments. The method makes use of the previously presented concept of the TM. A model is set up that provides an analytical description of the $B_{1}^{+}$ magnitude and transceive phase distribution around a wire‐like implant. In this model, the background field and the TM of the implant are described by a relatively small set of unknown parameters. The background field is described using a superposition of spherical and cylindrical harmonics; the TM is described using a previously introduced attenuated wave model. The analytical description can be used to fit measured $B_{1\;tot}^{+}$ and transceive phase distributions to assess the TM and TF of an elongated implant with MRI measurements. This MRI‐based method to measure the TF does not require hardware alterations to implant or scanner; neither does it rely on field distributions from simulations.

The method has been tested for 2 implant‐mimicking wires: a 20‐cm bare copper wire and a 20‐cm insulated copper wire with 10 mm of insulation stripped at both endings. Based on an in silica experiment, the method is able to determine the transfer function with correlation coefficients with respect to a gold standard above 0.94. Actual measurements likewise show a strong correlation with numerical simulations of the same setup and with each other. The maximum deviation in the estimated electric field around the tip of the implant based on the measured transfer function is 9.4% and 12.2% compared to the reference for the bare and insulated wire, respectively.

## Supporting information


**FIGURE S1** The TF and TM that follow from the fit of the simulated fields (b) and (bi) shown in Figures 3 and 4, respectively, corresponding to the straight wires under a 15° angle with the z‐axis
**FIGURE S2** The TF and TM that follow from the fit of the measured fields (b) and (bi) shown in Figures 8 and 9, respectively, corresponding to the straight wires under a 15° angle with the z‐axis
**FIGURE S3** The TF and TM that follow from the fit of the simulated fields (c) and (ci) shown in Figures 3 and 4, respectively, corresponding to the straight wires under a 30° angle with the z‐axis
**FIGURE S4** The TF and TM that follow from the fit of the measured fields (c) and (ci) shown in Figures 8 and 9, respectively, corresponding to the straight wires under a 30° angle with the z‐axis
**FIGURE S5** The TF and TM that follow from the fit of the simulated fields (d) and (di) shown in Figures 3 and 4, respectively, corresponding to the wires with a single bend
**FIGURE S6** The TF and TM that follow from the fit of the measured distributions (d) shown in Figure 8, corresponding to the bare wire with a single bend
**FIGURE S7** The TF and TM that follow from the fit of the simulated distributions (e) shown in Figure 3, corresponding to the bare wire with multiple bends
