# Supplementary material for: MRI‐based transfer function determination through the transfer matrix by jointly fitting the incident and scattered B1+ field
Source: Magn Reson Med. 2019 Oct 21;83(3):1081–95. doi: 10.1002/mrm.27974 (PMC6899904; doi:10.1002/mrm.27974)
Supplement: Supplementary file 1 — FIGURE S1 The TF and TM that follow from the fit of the simulated fields (b) and (bi) shown in Figures 3 and 4, respectively, corresponding to the straight wires under a 15° angle with the z‐axis FIGURE S2 The TF and TM that follow from the fit of the measured fields (b) and (bi) shown in Figures 8 and 9, respectively, corresponding to the straight wires under a 15° angle with the z‐axis FIGURE S3 The TF and TM that follow from the fit of the simulated fields (c) and (ci) shown in Figures 3 and 4, respectively, corresponding to the straight wires under a 30° angle with the z‐axis FIGURE S4 The TF and TM that follow from the fit of the measured fields (c) and (ci) shown in Figures 8 and 9, respectively, corresponding to the straight wires under a 30° angle with the z‐axis FIGURE S5 The TF and TM that follow from the fit of the simulated fields (d) and (di) shown in Figures 3 and 4, respectively, corresponding to the wires with a single bend FIGURE S6 The TF and TM that follow from the fit of the measured distributions (d) shown in Figure 8, corresponding to the bare wire with a single bend FIGURE S7 The TF and TM that follow from the fit of the simulated distributions (e) shown in Figure 3, corresponding to the bare wire with multiple bends [file MRM-83-1081-s001.docx]

**Supporting figures**

Supporting Information Figure S1. The TF and TM that follow from the fit of the simulated fields B and Bi shown in figures 3 and 4 respectively, corresponding to the straight wires under a 15 degrees angle with the z-axis.

Supporting Information Figure S2. The TF and TM that follow from the fit of the measured fields B and Bi shown in figures 8 and 9 respectively, corresponding to the straight wires under a 15 degrees angle with the z-axis.

Supporting Information Figure S3. The TF and TM that follow from the fit of the simulated fields C and Ci shown in figures 3 and 4 respectively, corresponding to the straight wires under a 30 degrees angle with the z-axis.

Supporting Information Figure S4. The TF and TM that follow from the fit of the measured fields C and Ci shown in figures 8 and 9 respectively, corresponding to the straight wires under a 30 degrees angle with the z-axis.

Supporting Information Figure S5. The TF and TM that follow from the fit of the simulated fields D and Di shown in figures 3 and 4 respectively, corresponding to the wires with a single bend.

Supporting Information Figure S6. The TF and TM that follow from the fit of the measured distributions D shown in figures 8, corresponding to the bare wire with a single bend.

Supporting Information Figure S7. The TF and TM that follow from the fit of the simulated distributions E shown in figures 3 corresponding to the bare wire with a multiple bends.
